# Complete genome sequence of *Cellulophaga algicola* type strain (IC166^T^)

**DOI:** 10.4056/sigs.1543845

**Published:** 2011-02-22

**Authors:** Birte Abt, Megan Lu, Monica Misra, Cliff Han, Matt Nolan, Susan Lucas, Nancy Hammon, Shweta Deshpande, Jan-Fang Cheng, Roxane Tapia, Lynne Goodwin, Sam Pitluck, Konstantinos Liolios, Ioanna Pagani, Natalia Ivanova, Konstantinos Mavromatis, Galina Ovchinikova, Amrita Pati, Amy Chen, Krishna Palaniappan, Miriam Land, Loren Hauser, Yun-Juan Chang, Cynthia D. Jeffries, John C. Detter, Evelyne Brambilla, Manfred Rohde, Brian J. Tindall, Markus Göker, Tanja Woyke, James Bristow, Jonathan A. Eisen, Victor Markowitz, Philip Hugenholtz, Nikos C. Kyrpides, Hans-Peter Klenk, Alla Lapidus

**Affiliations:** 1 DSMZ - German Collection of Microorganisms and Cell Cultures GmbH, Braunschweig, Germany; 2 DOE Joint Genome Institute, Walnut Creek, California, USA; 3 Los Alamos National Laboratory, Bioscience Division, Los Alamos, New Mexico, USA; 4 Biological Data Management and Technology Center, Lawrence Berkeley National Laboratory, Berkeley, California, USA; 5 Oak Ridge National Laboratory, Oak Ridge, Tennessee, USA; 6 HZI – Helmholtz Centre for Infection Research, Braunschweig, Germany; 7 University of California Davis Genome Center, Davis, California, USA; 8 Australian Centre for Ecogenomics, School of Chemistry and Molecular Biosciences,The University of Queensland, Brisbane, Australia

**Keywords:** aerobic, motile by gliding, Gram-negative, agarolytic, chemoorganotrophic, cold adapted enzymes, *Flavobacteriaceae*, GEBA

## Abstract

*Cellulophaga algicola* Bowman 2000 belongs to the family *Flavobacteriaceae* within the phylum '*Bacteroidetes*' and was isolated from *Melosira* collected from the Eastern Antarctic coastal zone. The species is of interest because its members produce a wide range of extracellular enzymes capable of degrading proteins and polysaccharides with temperature optima of 20-30°C. This is the first completed genome sequence of a member of the genus *Cellulophaga*. The 4,888,353 bp long genome with its 4,285 protein-coding and 62 RNA genes consists of one circular chromosome and is a part of the *** G****enomic* *** E****ncyclopedia of* *** B****acteria and* *** A****rchaea * project.

## Introduction

Strain IC166^T^ (= DSM 14237 = CIP 107446 = LMG 21425) is the type strain of *C. algicola,* which belongs to the family *Flavobacteriaceae* within the phylum '*Bacteroidetes*'. The strain was isolated from the surface of the chain-forming sea-ice diatom *Melosira* collected from the Eastern Antarctic coastal zone, and was described by Bowman in 2000 [1]. Currently, there are six species placed in the genus *Cellulophaga*, namely *C. algicola* [1], *C. baltica, C. fucicola*, *C. lytica* [2], *C. pacifica* [3] and *C. tyrosinoxydans* [4]. *C. lytica* is the type species of the genus *Cellulophaga* [2]. The generic name of the genus derives from the Neo Latin word '*cellulosum*' meaning 'cellulose' and the Greek word '*phagein*' meaning 'to eat', referring to an eater of cellulose. Here we present a summary classification and a set of features for *C. algicola* IC166^T^, together with the description of the complete genomic sequencing and annotation.

## Classification and features

A representative genomic 16S rRNA sequence of *C. algicola* was compared using NCBI BLAST under default settings (e.g., considering only the high-scoring segment pairs (HSPs) from the best 250 hits) with the most recent release of the Greengenes database [5] and the relative frequencies, weighted by BLAST scores, of taxa and keywords (reduced to their stem [6]) were determined. The five most frequent genera were *Cellulophaga* (39.5%), *Maribacter* (7.8%), *Flavobacterium* (5.6%), *Cytophaga* (5.4%) and *Formosa* (4.7%) (135 hits in total). Regarding the 21 hits to sequences from members of the species, the average identity within HSPs was 95.8%, whereas the average coverage by HSPs was 94.9%. Regarding the 16 hits to sequences from other members of the genus, the average identity within HSPs was 94.7%, whereas the average coverage by HSPs was 94.7%. Among all other species, the one yielding the highest score was *C. baltica*, which corresponded to an identity of 98.1% and a HSP coverage of 97.8%. The highest-scoring environmental sequence was GU452686 ('sediments coast oil polluted Black Sea coastal sediment clone 70SZ2'), which showed an identity of 96.5% and a HSP coverage of 98.1%. The five most frequent keywords within the labels of environmental samples which yielded hits were 'marin' (4.7%), 'water' (4.3%), 'sediment' (4.3%), 'sea' (3.5%) and 'coastal' (2.6%) (115 hits in total). Environmental samples which yielded hits of a higher score than the highest scoring species were not found.

The environmental samples database (env_nt) contains the marine metagenome clone ctg_1101667042524 (AACY022635173) isolated from Sargasso Sea near Bermuda, sharing 92% identity with IC166^T^ [7] (as of January 2011).

**Figure 1:**
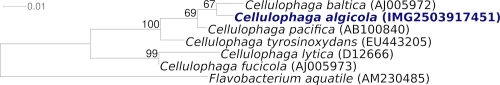
Phylogenetic tree highlighting the position of *C. algicola* IC166^T^ relative to the other type strains within the family *Flavobacteriaceae*. The tree was inferred from 1,458 aligned characters [8,9] of the 16S rRNA gene sequence under the maximum likelihood criterion [10] and rooted in accordance with the current taxonomy. The branches are scaled in terms of the expected number of substitutions per site. Numbers above branches are support values from 350 bootstrap replicates [11] if larger than 60%. Lineages with type strain genome sequencing projects registered in GOLD [12] are shown in blue, published genomes in bold.

Figure 1 shows the phylogenetic neighborhood of *C. algicola* IC166^T^ in a 16S rRNA based tree. The sequences of the five 16S rRNA gene copies in the genome differ from each other by up to two nucleotides, and differ by up to 14 nucleotides from the previously published 16S rRNA sequence (AF001366), which contains nine ambiguous base calls.

**Figure 2:**
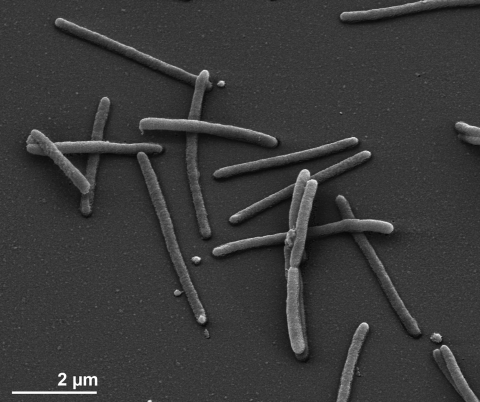
Scanning electron micrograph of *C. algicola* IC166^T^

The cells of *C. algicola* are generally rod-shaped with rounded or tapered ends with cell lengths and widths ranging from 1.5 to 4 and 0.4 to 0.5 µm, respectively (Figure 2 and Table 1). *C. algicola* is motile by gliding [1]. Colonies on marine 2216 agar have yellow-orange pigmentation and a compact center, with a spreading edge possessing lighter pigmentation. Their consistency is slimy and they are slightly sunken into the agar [1]. Flexirubin pigments are not formed. *C. algicola* grows between 0.5 and 10% NaCl, with the best growth in the presence of about 2% NaCl. The temperature range for growth is between -2°C and 28°C, with an optimum between 15-20°C on solid media and at about 20-25°C in liquid media [1]. The optimal pH for growth is about 7.5 [1].

**Table 1:** Classification and general features of *C. algicola* IC166^T^ according to the MIGS recommendations [13].

MIGS ID	Property	Term	Evidence code
	Current classification	Domain *Bacteria*	TAS [14]
		Phylum *Bacteroidetes*	TAS [15,16]
		Class *Flavobacteria*	TAS [17]
		Order '*Flavobacteriales*'	TAS [15]
		Family *Flavobacteriaceae*	TAS [18-21]
		Genus *Cellulophaga*	TAS [2]
		Species *Cellulophaga algicola*	TAS [1]
		Type strain IC166	TAS [1]
	Gram stain	negative	TAS [1]
	Cell shape	rod-shaped	TAS [1]
	Motility	motile by gliding	TAS [1]
	Sporulation	none	TAS [1]
	Temperature range	-2 °C – 28°C	TAS [1]
	Optimum temperature	20°C	TAS [1]
	Salinity	0.5-10% NaCl	TAS [1]
MIGS-22	Oxygen requirement	aerobic	TAS [1]
	Carbon source	carbohydrates	TAS [1]
	Energy source	chemoheterotroph	TAS [1]
MIGS-6	Habitat	sea ice diatoms, macrophyte surfaces	TAS [1]
MIGS-15	Biotic relationship	free-living	NAS
MIGS-14	Pathogenicity	none	NAS
	Biosafety level	1	TAS [22]
	Isolation	surfaces of Antarctic algae	TAS [1]
MIGS-4	Geographic location	eastern Antarctic coastal zone	TAS [1]
MIGS-5	Sample collection time	1996	NAS
MIGS-4.1	Latitude	not reported	NAS
MIGS-4.2	Longitude	not reported	NAS
MIGS-4.3	Depth	not reported	NAS
MIGS-4.4	Altitude	not reported	NAS

Evidence codes - IDA: Inferred from Direct Assay (first time in publication); TAS: Traceable Author Statement (i.e., a direct report exists in the literature); NAS: Non-traceable Author Statement (i.e., not directly observed for the living, isolated sample, but based on a generally accepted property for the species, or anecdotal evidence). These evidence codes are from of the Gene Ontology project [23]. If the evidence code is IDA, then the property was directly observed by one of the authors or an expert mentioned in the acknowledgements.

The organism is strictly aerobic and chemoorganotrophic [1]. *C. algicola* can hydrolyze agar, starch, gelatine, carboxymethylcellulose (CMC), casein, Tween 80, tributyrin and L-tyrosine, but not urate, xanthine or dextran, when grown in presence of 1% L-tyrosine a reddish-brown diffusible pigment is formed [1]. Nitrate reduction is positive, whereas denitrification, H_2_S production and indole production are negative [1,18]. Acid is formed oxidatively from D-galactose, D-glucose, D-fructose, sucrose, cellobiose, lactose and mannitol. Strain IC166^T^ is sensitive to ampicillin, streptomycin and carbenicillin and shows resistance to tetracycline [3].

### Chemotaxonomy

The fatty acid profile of seven Antarctic strains, including strain IC166^T^, was analyzed by Bowman in 2000 [1]. The hypothetical median representative of the Antarctic isolates was published. The predominant cellular fatty acids of these seven strains were branched-chain saturated and unsaturated fatty acids and straight-chain saturated and mono-unsaturated fatty acids, namely *iso*-C_15:0_ (7.5%), *iso*-C_15:1ω10c_ (7.5%), *iso* -C_17:1ω7c_ (6.1%), C_15:0_ (14.3%), C_16:1ω7c_ (19.2%), *iso* -C_15:0 3-OH_ (8.6%), *iso*-C_16:0 3-OH_ (6.5%) and *iso* -C_17:0 3-OH_ (4.5%) [1]. The isoprenoid quinones of *C. algicola* were not determined, but for *C*. *pacifica* the presence of MK-6 as the major lipoquinone was described [3]. Polar lipids not have been studied.

## Genome sequencing and annotation

### Genome project history

This organism was selected for sequencing on the basis of its phylogenetic position [24], and is part of the *** G****enomic* *** E****ncyclopedia of* *** B****acteria and* *** A****rchaea * project [25]. The genome project is deposited in the Genomes OnLine Database [12] and the complete genome sequence is deposited in GenBank. Sequencing, finishing and annotation were performed by the DOE Joint Genome Institute (JGI). A summary of the project information is shown in Table 2.

**Table 2:** Genome sequencing project information

**MIGS ID**	**Property**	**Term**
MIGS-31	Finishing quality	Finished
MIGS-28	Libraries used	Three genomic libraries: one 454 pyrosequence standard library, one 454 PE library (12 kb insert size), one Illumina library
MIGS-29	Sequencing platforms	Illumina GAii, 454 GS FLX Titanium
MIGS-31.2	Sequencing coverage	146.0 × Illumina; 53.5 × pyrosequence
MIGS-30	Assemblers	Newbler version 2.0.00.20-PostRelease-10-28-2008-g-3.4.6, Velvet version 0.7.63, phrap version SPS D 4.24
MIGS-32	Gene calling method	Prodigal 1.4, GenePRIMP
	INSDC ID	CP002453
	Genbank Date of Release	January 18, 2011
	GOLD ID	Gc01592
	NCBI project ID	41529
	Database: IMG-GEBA	2503904003
MIGS-13	Source material identifier	DSM 14237
	Project relevance	Tree of Life, GEBA

### Growth conditions and DNA isolation

*C. algicola* IC166^T^, DSM 14237, was grown in DSMZ medium 514 (BACTO marine broth) [26] at 15°C. DNA was isolated from 0.5-1 g of cell paste using MasterPure Gram-positive DNA purification kit (Epicentre MGP04100) following the standard protocol as recommended by the manufacturer with modification st/DL for cell lysis as described in Wu *et al*. [25]. DNA is available through the DNA Bank Network [27].

### Genome sequencing and assembly

The genome was sequenced using a combination of Illumina and 454 sequencing platforms. All general aspects of library construction and sequencing can be found at the JGI website [28]. Pyrosequencing reads were assembled using the Newbler assembler version 2.3-PreRelease-09-14-2009-bin (Roche). The initial Newbler assembly consisting of 128 contigs in two scaffolds was converted into a phrap assembly by [29] making fake reads from the consensus, to collect the read pairs in the 454 paired end library. Illumina GAii sequencing data (710 Mb) was assembled with Velvet [30] and the consensus sequences were shredded into 1.5 kb overlapped fake reads and assembled together with the 454 data. The 454 draft assembly was based on 263.4Mb 454 draft data and all of the 454 paired end data. Newbler parameters are -consed -a 50 -l 350 -g -m -ml 20. The Phred/Phrap/Consed software package [29] was used for sequence assembly and quality assessment in the subsequent finishing process. After the shotgun stage, reads were assembled with parallel phrap (High Performance Software, LLC). Possible mis-assemblies were corrected with gapResolution [28], Dupfinisher [31], or sequencing cloned bridging PCR fragments with subcloning or transposon bombing (Epicentre Biotechnologies, Madison, WI). Gaps between contigs were closed by editing in Consed, by PCR and by Bubble PCR primer walks (J.-F.Chang, unpublished). A total of 1,054 additional reactions and three shatter libraries were necessary to close gaps and to raise the quality of the finished sequence. Illumina reads were also used to correct potential base errors and increase consensus quality using a software Polisher developed at JGI [32]. The error rate of the completed genome sequence is less than 1 in 100,000. Together, the combination of the Illumina and 454 sequencing platforms provided 199.5 × coverage of the genome. The final assembly contained 697,305 pyrosequence and 20,331,123 Illumina reads

### Genome annotation

Genes were identified using Prodigal [33] as part of the Oak Ridge National Laboratory genome annotation pipeline, followed by a round of manual curation using the JGI GenePRIMP pipeline [34]. The predicted CDSs were translated and used to search the National Center for Biotechnology Information (NCBI) nonredundant database, UniProt, TIGR-Fam, Pfam, PRIAM, KEGG, COG, and InterPro databases. Additional gene prediction analysis and functional annotation was performed within the Integrated Microbial Genomes - Expert Review (IMG-ER) platform [35].

### Genome properties

**Figure 3:**
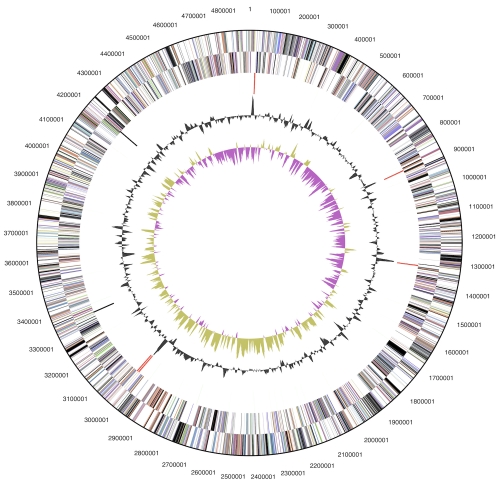
Graphical circular map of the chromosome. From outside to the center: Genes on forward strand (color by COG categories), Genes on reverse strand (color by COG categories), RNA genes (tRNAs green, rRNAs red, other RNAs black), GC content, GC skew.

The genome consists of a 4,888,353 bp long chromosome with a GC content of 33.8% (Table 3 and Figure 3). Of the 4,347 genes predicted, 4,285 were protein-coding genes, and 62 RNAs; 122 pseudogenes were also identified. The majority of the protein-coding genes (59.5%) were assigned with a putative function while the remaining ones were annotated as hypothetical proteins. The distribution of genes into COGs functional categories is presented in Table 4.

**Table 3:** Genome Statistics

**Attribute**	**Value**	**% of Total**
Genome size (bp)	4,888,353	100.00%
DNA coding region (bp)	4,301,528	88.00%
DNA G+C content (bp)	1,650,610	33.77%
Number of replicons	1	
Extrachromosomal elements	0	
Total genes	4,347	100.00%
RNA genes	62	1.43%
rRNA operons	5	
Protein-coding genes	4,285	98.57%
Pseudo genes	122	2.81%
Genes with function prediction	2,587	59.51%
Genes in paralog clusters	698	16.06%
Genes assigned to COGs	2,539	58.41%
Genes assigned Pfam domains	2,822	64.92%
Genes with signal peptides	1,220	28.07%
Genes with transmembrane helices	1,010	23.23%
CRISPR repeats	0	

**Table 4:** Number of genes associated with the general COG functional categories

Code	value	%age	Description
J	160	5.8	Translation, ribosomal structure and biogenesis
A	0	0.0	RNA processing and modification
K	174	6.3	Transcription
L	147	5.4	Replication, recombination and repair
B	1	0.0	Chromatin structure and dynamics
D	20	0.7	Cell cycle control, cell division, chromosome partitioning
Y	0	0.0	Nuclear structure
V	63	2.3	Defense mechanisms
T	167	6.1	Signal transduction mechanisms
M	239	8.7	Cell wall/membrane/envelope biogenesis
N	7	0.3	Cell motility
Z	0	0.0	Cytoskeleton
W	0	0.0	Extracellular structures
U	41	1.5	Intracellular trafficking, secretion, and vesicular transport
O	99	3.6	Posttranslational modification, protein turnover, chaperones
C	135	4.9	Energy production and conversion
G	172	6.3	Carbohydrate transport and metabolism
E	208	7.6	Amino acid transport and metabolism
F	70	2.6	Nucleotide transport and metabolism
H	131	4.8	Coenzyme transport and metabolism
I	97	3.5	Lipid transport and metabolism
P	174	6.3	Inorganic ion transport and metabolism
Q	52	1.9	Secondary metabolites biosynthesis, transport and catabolism
R	345	12.6	General function prediction only
S	247	9.0	Function unknown
-	1,808	41.6	Not in COGs

## Insights from genome sequence

A closer look on the genome sequence of strain IC166^T^ revealed a set of genes which might be responsible for the yellow-orange color of *C. algicola* cells by encoding enzymes that are involved in the synthesis of carotenoids. Carotenoids are produced by the action of geranylgeranyl pyrophosphate synthase (Celal_1770), phytoene synthase (Celal_2446), phytoene desaturase (Celal_2447), lycopene cyclase (Celal_1771) and carotene hydroxylase (Celal_2445). Geranylgeranyl pyrophosphate synthases start the biosynthesis of carotenoids by combining farnesyl pyrophosphate with C_5_ isoprenoid units to C_20_-molecules, geranylgeranyl pyrophosphate. The phytoene synthase catalyzes the condensation of two geranylgeranyl pyrophosphate molecules followed by the removal of diphosphate and a proton shift leading to the formation of phytoene. Sequential desaturation steps are conducted by the phytoene desaturase followed by cyclisation of the ends of the molecules catalyzed by the lycopene cyclase [36].

Strain IC166^T^ produces a wide range of extracellular enzymes degrading proteins and polysaccharides. These enzymes are cold adapted, they have temperature optima between 15-30°C and can tolerate temperatures below 0°C [37]. For that reason they are of special interest for industrial and biotechnical applications. *C. algicola* like the other members of the genus *Cellulophaga*, cannot hydrolyze filter paper or cellulose in its crystalline form, though they can hydrolyze the soluble cellulose derivative carboxymethylcellulose (CMC). The genome sequence of strain IC166^T^ revealed the presence of three cellulases (Celal_0025, Celal_2753, Celal_3912), probably responsible for the hydrolysis of CMC. In addition two β-glucosidases (Celal_0470, Celal_1802) were identified in the genome, catalyzing the break down of the glycosidic β**-**1,4 bond between two glucose molecules in cellobiose.

The IC166^T^ genome contains 22 genes coding for sulfatases, which are located in close proximity to glycoside hydrolase genes suggesting that sulfated polysaccharides may be used as substrates. α-L-fucoidan could be a substrate, as five α-L-fucosidases (Celal_2459, Celal_2466, Celal_2469, Celal_2470, Celal_2473) are located in close proximity to three sulfatases (Celal_2464, Celal_2468, Celal_2472). Sakai and colleagues report the existence of intracellular α-L-fucosidases and sulfatases, which enable '*Fucophilus fucoidanolyticus*' to degrade fucoidan [38]. This fucoidan degrading ability could be also shared by *Coraliomargarita akajimensis*, as the annotation of the genome sequence revealed the existence of 49 sulfatases and twelve α-L-fucosidases [39]. In addition, three β-agarases (Celal_2463, Celal_2494, Celal_3979) were identified, with two of them located in the above mentioned region, which is rich in genes encoding glycoside hydrolases and sulfatases.
